# 5′-UTR allelic variants and expression of the lycopene-ɛ-cyclase LCYE gene in maize (Zea mays L.) inbred lines of Russian selection

**DOI:** 10.18699/VJGB-23-53

**Published:** 2023-09

**Authors:** D.Kh. Arkhestova, B.R. Shomakhov, A.V. Shchennikova, E.Z. Kochieva

**Affiliations:** Institute of Bioengineering, Federal Research Center “Fundamentals of Biotechnology” of the Russian Academy of Sciences, Moscow, Russia Institute of Agriculture – Branch of the Federal Scientific Center “Kabardino-Balkarian Scientific Center of the Russian Academy of Sciences”, Nalchik, Russia; Institute of Agriculture – Branch of the Federal Scientific Center “Kabardino-Balkarian Scientific Center of the Russian Academy of Sciences”, Nalchik, Russia; Institute of Bioengineering, Federal Research Center “Fundamentals of Biotechnology” of the Russian Academy of Sciences, Moscow, Russia; Lomonosov Moscow State University, Moscow, Russia

**Keywords:** Zea mays L., maize inbred lines, lycopene-ε-cyclase, LCYE alleles, gene expression, Zea mays L., инбредные линии кукурузы, ликопин-ε-циклаза, аллели LCYE, экспрессия гена

## Abstract

In breeding, biofortification is aimed at enriching the edible parts of the plant with micronutrients. Within the framework of this strategy, molecular screening of collections of various crops makes it possible to determine allelic variants of genes, new alleles, and the linkage of allelic variants with morphophysiological traits. The maize (Zea mays L.) is an important cereal and silage crop, as well as a source of the main precursor of vitamin A – β-carotene, a derivative of the β,β-branch of the carotenoid biosynthesis pathway. The parallel β,ε-branch is triggered by lycopene-ε-cyclase LCYE, a low expression of which leads to an increase in provitamin A content and is associated with the variability of the 5’-UTR gene regulatory sequence. In this study, we screened a collection of 165 maize inbred lines of Russian selection for 5’- UTR LCYE allelic variants, as well as searched for the dependence of LCYE expression levels on the 5’-UTR allelic variant in the leaves of 14 collection lines. 165 lines analyzed were divided into three groups carrying alleles A2 (64 lines), A5 (31) and A6 (70), respectively. Compared to A2, allele A5 contained two deletions (at positions -267– 260 and -296–290 from the ATG codon) and a G251→T substitution, while allele A6 contained one deletion (-290–296) and two SNPs (G251→T, G265→T). Analysis of LCYE expression in the leaf tissue of seedlings from accessions of 14 lines differing in allelic variants showed no associations of the 5’-UTR LCYE allele type with the level of gene expression. Four lines carrying alleles A2 (6178-1, 6709-2, 2289-3) and A5 (5677) had a significantly higher level of LCYE gene expression (~0.018–0.037) than the other 10 analyzed lines (~0.0001–0.004), among which all three allelic variants were present.

## Abstract (ru)

Селекционная биофортификация направлена на обогащение съедобных частей растения микронутриентами. В рамках данной стратегии молекулярный скрининг коллекций различных культур позволяет определять аллельные варианты генов, новые аллели и сцепленность аллельных вариантов с морфофизиологическими признаками. Кукуруза Zea mays L. является важной зерновой и силосной культурой, а также источником основного предшественника витамина А – β-каротина, производного β,β-ветви пути биосинтеза каротиноидов. Параллельная β,ε-ветвь запускается ликопин-ε-циклазой LCYE, низкая экспрессия которой приводит к росту содержания провитамина А и связана с вариабельностью регуляторной последовательности 5’-UTR гена. В настоящем исследовании проведены скрининг коллекции 165 инбредных линий кукурузы российской селекции на варианты аллелей 5’-UTR LCYE, а также поиск зависимости уровня экспрессии гена LCYE от аллельного варианта 5’-UTR в листьях 14 коллекционных линий. Проанализированные 165 линий разделились на три группы, несущие аллели А2 (64 линии), А5 (31) и А6 (70). В сравнении с А2, аллель А5 содержал две делеции (в позициях -267–260 и -296–290 от ATG-кодона) и замену G251→T, тогда как аллель А6 – одну делецию (-290–296) и две замены (G251→T, G265→T). Анализ экспрессии гена LCYE в листовой ткани проростков образцов 14 линий, различающихся аллель- ными вариантами, показал отсутствие ассоциаций варианта аллеля 5’-UTR LCYE с уровнем экспрессии гена. Четыре линии, несущие аллели А2 (образцы 6178-1, 6709-2, 2289-3) и А5 (образец 5677), имели значительно более высокий уровень экспрессии гена LCYE (~0.018–0.037) по сравнению с остальными десятью проанализированными линиями (~0.0001–0.004), среди которых были представлены все три аллельных варианта.

## Introduction

Maize Zea mays L. is an important world crop. Climatic conditions in Russia favor the predominant cultivation of corn for silage (immature cobs, leaves and stems), which makes up about 50 % of the dry matter of the main feed for farm animals (Cabiddu et al., 2019; Graulet et al., 2019; Mitani et al., 2021). As a grain crop, corn is grown only in the southern regions of Russia. According to the Ministry of Agriculture, 1.4 million tons of grain were harvested in 2021, which is ~50 times less compared to wheat (https://mcx.gov.ru/press-service/news/ sbor-zernovykh-v-rossii-dostig-100-mln-tonn/).

Both grain and silage of maize are considered important sources of antioxidants, including provitamin A, represented by three carotenoid compounds: β-carotene (provides two units of retinol – active vitamin A, when oxidatively broken down), β-cryptoxanthin (provides one unit of retinol, but with greater bioavailability than β-carotene) and α-carotene (one unit of retinol) (LaPorte et al., 2022). In addition to dietary significance, enrichment with β-carotene and β-cryptoxanthin contributes to an essential reduction in aflatoxin contamination of corn grain (Suwarno et al., 2019). In the grain of the most popular varieties and hybrids, according to various data, carotenoids range from 9.55 to 62.96 μg/g (Trono, 2019), while in the leaves their content is already about 200 μg/g (Li et al., 2008; Suwarno et al., 2019).

**Fig. 1.**
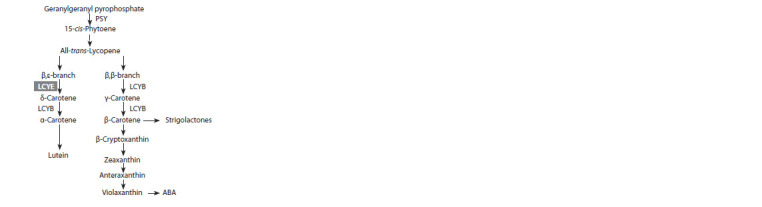
A simplified scheme of xanthophylls biosynthesis. Phytoene synthase PSY catalyzes the synthesis of phytoene, from which translycopene is formed as a result of several successive reactions. Further, the metabolic pathway is divided into β,β- and β,ε-branches, which lead to the production of β-carotene and α-carotene, respectively, and then β-cryptoxanthin and xanthophylls – zeaxanthin, antheraxanthin, violaxanthin (β,β-carotenoids) and lutein (β,ε-carotenoids). The modification of β-carotene and violaxanthin under the action of carotenoid-cleaving dioxygenases leads to the synthesis of apocarotenoids – strigolactones and abscisic acid (ABA), respectively.

β-Carotene and β-cryptoxanthin are products of the β,β- branch of the carotenoid biosynthetic pathway (Fig. 1): lycopene-β-cyclase (LCYB) catalyzes the formation of β-ionone rings at both ends of the all-trans-lycopene molecule with the formation of β-carotene, the hydroxylation of which leads to the synthesis of xanthophylls, including β-cryptoxanthin (Rosas-Saavedra, Stange, 2016). The α-carotene molecule, a product of the β,ε-branch triggered by lycopene-ε-cyclase (LCYE) (see Fig. 1), is characterized by a β-ring at one end and an ε-ring at the other end of the isoprenoid chain (Rosas-Saavedra, Stange, 2016). A signature of the predominance of the β,β- or β,ε-branch is the orange or light yellow, respectively, color of the corn grain (Harjes et al., 2008; Babu et al., 2013; Zunjare et al., 2018).

Maize breeding for provitamin A biofortification uses the LCYE gene, as well as the β-carotene hydroxylase 1 (CrtRB1) gene, which catalyzes the conversion of β-carotene to β-cryptoxanthin (LaPorte et al., 2022). A decrease in the expression level of the first, second, or both genes simultaneously leads to a shift in the metabolic pathway towards the biosynthesis of β-carotene as the most promising source of provitamin A (Harjes et al., 2008; Yan et al., 2010; Muthusamy et al., 2014; Liu et al., 2015; Zunjare et al., 2018; LaPorte et al., 2022).

One of the main conditions for successful breeding is the availability of donors of allelic variants linked to the desired economically valuable traits. Maize accessions, which are characterized by low grain expression of LCYE and/or CrtRB1 genes, are used in breeding, including provitamin A biofortification (Pixley et al., 2013; Muthusamy et al., 2014; Liu et al., 2015; Menkir et al., 2017; Prasanna et al., 2020). It has been shown that a reduced level of LCYE transcripts may be associated with polymorphisms in the 5′-UTR sequence of the gene (Harjes et al., 2008; Babu et al., 2013; Zunjare et al., 2018).

With all the promise of LCYE alleles in maize biofortification, as well as the widespread use of maize for silage, studies of the gene activity are limited to corn grain and barely touch upon photosynthetic organs. Previously, we have shown an inverse relationship between the content of β-carotene and the level of LCYE gene expression in the leaf tissue of maize seedlings (Arkhestova et al., 2022).

In the study, we assessed the correlations between the level of LCYE expression and allelic variants of the 5′-UTR regulatory region of the gene in a collection of 165 inbred maize lines. We also analyzed the relationship between the level of expression and the allelic variant of the lycopene-ɛ-cyclase gene in the leaves of 14 accessions differing in the 5ʹ-UTR LCYE alleles. The maize lines used in the work were obtained by breeders of two organizations in the Kabardino-Balkarian

Republic (KBR, North Caucasian Federal District), which, due to climatic conditions, ranks first in the Russian Federation in the cultivation of corn, according to the allocated sown area (Rosstat data for 2021; https://rosstat.gov.ru/storage/mediabank/ Census_agr_2021.pdf).

## Materials and methods

Accessions of 165 Z. mays inbred lines from two breeding organizations (JSC “OTBOR” and the Institute of Agriculture KBSC RAS) were used for the study; the lines are currently being tested and are listed in the work under the numbers assigned to them by the breeders (see Supplementary Material)1.

Supplementary Materials are available in the online version of the paper: http://vavilov.elpub.ru/jour/manager/files/Suppl_Arkhestova_Engl_27_5.pdf


**Fig. 2.**
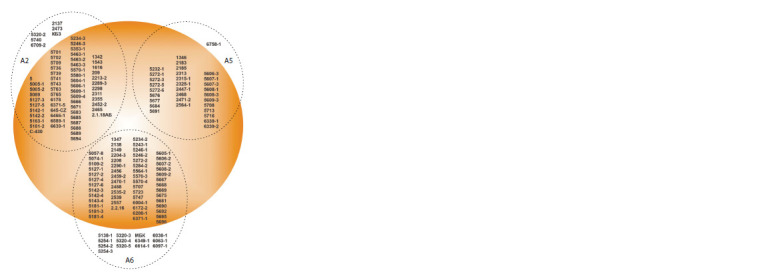
Distribution of 165 Zea mays L. inbred lines (domestic breeding) used in the study according to grain color and 5’-UTR allelic variants of the LCYE gene (delimited by ellipses, within which the corresponding allele is indicated – A2, A5, or A6). White-grain accessions are placed in the unpainted part of the ellipses; lines with grain coloring in various shades of yellow, orange and red are located in the colored part.

The seed material of plants grown in the field in 2022 (KBR, Russia) was kindly provided by the JSC “OTBOR” (KBR, Russia) and the Institute of Agriculture of the Branch of the Kabardino-Balkarian Scientific Center of the Russian Academy of Sciences (IA KBSC RAS, KBR, Russia). According to the originators (JSC “OTBOR”, IA KBSC RAS), the lines differ in grain color (Fig. 2, see Suppl. Material). Germinated grains were grown until the 4th true leaf appeared in moist soil under controlled conditions (23 °С/25 °С, 16/8 h day/night) of the experimental climate control facility in the Institute of Bioengineering (Research Center of Biotechnology, Russian Academy of Sciences). Leaf material was collected and used for analysis of LCYE allelic variants and expression.

To identify allelic variants, genomic DNA was isolated from the leaf material according to (Filyushin et al., 2023) and used as a template for PCR amplification of the 5ʹ-UTR region of the LCYE gene under the following conditions: initial denaturation (5 min, 95 °С), 32 cycles (denaturation 1 min, 95 °С; annealing 30 s, 60 °С; synthesis 45 s, 72 °С). Amplification primer sequences were: F2 (5′-AAGCATCCGACCAAAATAACAG-3′) and R2 (5′-GAGAGGGAGACGACGAGACAC-3′) (Harjes et al., 2008). The generated fragments purified from the gel (ZymocleanTM Gel DNA Recovery Kit, ZymoResearch, USA) were sequenced (primer F2) on an ABI 310 Capillary DNA Analyzer (Applied Biosystems, USA; Core Facility Use Bioengineering, Russian Academy of Sciences). Structural analysis was performed using NCBI-BLAST (https://blast. ncbi.nlm.nih.gov/Blast.cgi) and MEGA 7.0 (Kumar et al., 2016).

To analyze gene expression, total RNA was isolated from 50–100 mg of leaf tissue using the RNeasy Plant Mini Kit (QIAGEN, Germany), purified from genomic DNA impurities (RNase free DNasy set, QIAGEN), and used for cDNA synthesis (GoScriptTM Reverse Transcription System, Promega, USA). RNA quality was assessed by electrophoresis in 1.5 % agarose gel. The concentration of RNA and cDNA preparations was determined fluorimetrically using Qubit 4 (Thermo Fisher Scientific, USA) and reagents Qubit RNA HS Assay Kit and Qubit DS DNA HS Assay Kit (Invitrogen, USA).

The level of transcripts of the lycopene-ɛ-cyclase gene LCYE in the leaves of maize seedlings was determined by quantitative (q) real-time (RT) PCR (qRT-PCR). The data were normalized to the level of Z. mays polyubiquitin gene transcripts (NM_001329666.1; primers ZmUBI-rtF 5ʹ-ATCGTGGTTGTGGCTTCGTTG-3ʹ and ZmUBIr t R 5 ʹ - G C T G C A G A A G A G T T T T G G G TA C A - 3 ʹ ) .3 ng of cDNA, cDNA-specific primers (ZmLcyE-F 5ʹ-TTTACGTGCAAATGCAGTCAA-3ʹ; ZmLcyE-R 5ʹ-TGACTCTGAAGCTAGAGAAAG-3ʹ), kit “Reaction mixture for real-time PCR in the presence of SYBR GreenI and ROX” (Sintol, Russia), and thermal cycler CFX96 Real-Time PCR Detection System (Bio-Rad Laboratories, USA) were used for the reaction. The reactions were carried out in three technical and two biological replicates under the following conditions: preliminary denaturation (5 min, 95 °C); 40 cycles (15 s, 95 °C; 50 s, 62°C).

qRT-PCR results were statistically processed using GraphPad Prism v.8 (GraphPad Software Inc., USA; https:// www.graphpad.com/scientific-software/prism/). Data were expressed as mean with standard deviation (±SD) based on three technical and two biological replicates. The t-test was used to assess the significance of differences in gene expression between maize lines (p < 0.05 indicates statistical significance of differences).

## Results

The study was focused on the characterization of the allelic variability of the LCYE gene 5ʹ-UTR sequence in maize inbred lines of domestic selection, as well as the analysis of the level of gene transcripts in the leaf tissue of seedlings of lines that differ in allelic variants of the 5ʹ-UTR of the LCYE gene.

**Fig. 3.**
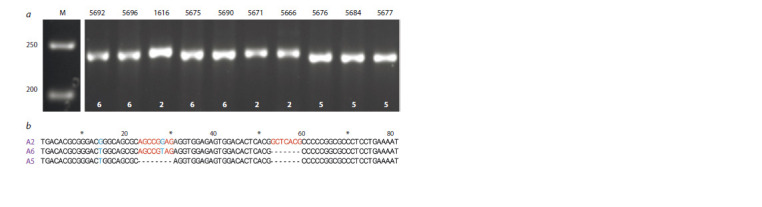
An example of electrophoretic separation of PCR amplified fragments (a) corresponding to the 5’-UTR LCYE allelic variants A2 (248 bp), A5 (233 bp), and A6 (240 bp) in a 2.5 % agarose gel (M is the length marker Thermo Fisher GeneRuler 50 bp) and comparative alignment of the variable region of the A2, A5, and A6 alleles (b). Indels in red, SNPs in blue.

To determine allelic variants of the lycopene-ɛ-cyclase gene, amplification and sequencing of the 5ʹ-UTR region of the LCYE gene was performed (Fig. 3). Expected fragment sizes: alleles A2 (248 bp, according to Harjes et al., 2008), A5 (233 bp, according to Arkhestova et al., 2023) and A4 (993 bp, according to Harjes et al., 2008).

As a result, no A4 variants were found, while alleles A2 and A5 were shown to be present in the analyzed accessions. In addition to these variants described earlier, a new, uncharacterized A6 allele (240 bp) was identified (see Fig. 3). In total, out of the analyzed 165 maize accessions, 64 lines contained the A2 allele, the smallest number of accessions (31) contained the A5 allele, and the largest number of accessions (70) contained the A6 allele (see Fig. 2).

It was determined that, unlike A2, 5ʹ-UTR of the A5 allele contains two deletions (at positions ˗267–260 and ˗296–290 from the ATG codon), while the new A6 allele has only one of these deletions (at position ˗296–290 from the ATG codon) (see Fig. 3). In addition, allele-specific single nucleotide substitutions were found in comparison with A2: G251→T (in the sequence of A5 and A6); G265→T (only for A6) (see Fig. 3). The positions of deletions and substitutions are given in accordance with the LCYE gene sequence available in the NCBI database (NCBI Gene ID OK032387.1).

**Fig. 4.**
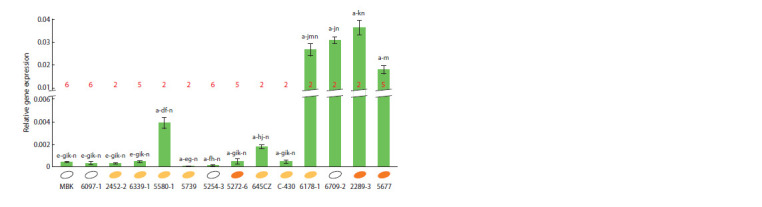
Relative level of LCYE gene expression in the leaf tissue of seedlings of 14 maize inbred lines. The letters a–n indicate a significant difference (p <0.05) of a particular gene expression value from the values for other accessions (lines 1–14 correspond to letters a–n). The allelic variant (A2 – 2, A5 – 5, or A6 – 6) is indicated in red for each accession. The grain color (white, bright yellow or orange) is shown by a colored ellipse next to the accession name.

Variants of the A2/A5/A6 alleles were found in accessions with white (59/30/57) and pigmented (5/1/13) grain, respectively. In order to understand whether the 5ʹ-UTR LCYE allele (A2, A5, or A6) is associated with the level of LCYE gene transcripts in photosynthetic tissue, LCYE expression was analyzed in the leaf tissue of 14 lines differing in allelic variants (Fig. 4). Accessions for qRT-PCR were selected based on two assumptions. First, all three types of alleles (A2, A5, and A6) of the LCYE g

Considering the qRT-PCR data, the analyzed accessions were clearly divided into two groups, which significantly differed in the level of LCYE gene expression (see Fig. 4). The first group combined four lines with a high expression level (~0.018–0.037), which was ~4.5–370.0 times higher than in ten accessions of the second group (~0.0001–0.004). Among A2 A6 A5 * 20 * 40 * 60 * 80 6 250 200 а M b 5692 5696 1616 5675 5690 5671 5666 5676 5684 5677 6 2 6 6 2 2 5 5 5 Fig. 3. An example of electrophoretic separation of PCR amplified fragments (a) corresponding to the 5’-UTR LCYE allelic variants A2 (248 bp), A5 (233 bp), and A6 (240 bp) in a 2.5 % agarose gel (M is the length marker Thermo Fisher GeneRuler 50 bp) and comparative alignment of the variable region of the A2, A5, and A6 alleles (b). Indels in red, SNPs in blue. the four lines with high expression, three (6178-1, 6709-2, and 2289-3) carried the A2 allele and one (5677) carried the A5 allele. At the same time, the level of LCYE transcripts in line 5677 (A5) was ~1.5–2.0 times lower than in three lines with the A2 allele (see Fig. 4).

In the group of lines with low gene expression, all three allelic variants of 5ʹ-UTR LCYE were present (see Fig. 4). This group included all three accessions with the A6 allele taken for analysis (MBK, 6097-1, 5254-3). Against the background of low gene activity in the group, lines 5580-1 and 645CZ (allelic variant A2) were characterized by increased activity of the LCYE gene (see Fig. 4).

It should be noted that lines carrying the A2 or A5 alleles were present both in the first and second groups (see Fig. 4).

Thus, using 14 lines representing all three variants of the 5ʹ-UTR LCYE allele as an example, it was shown that there was no dependence of the level of LCYE gene transcripts on the allele (A2, A5, or A6) in the leaf photosynthetic tissue.

## Discussion

Over the past decades, one of the most promising breeding trends has been biofortification (a strategy to improve the nutritional quality of cultivated plants by breeding methods using a number of biotechnologies) aimed at enriching the edible parts of the plant with micronutrients (vitamins, minerals and trace elements) (Medina-Lozano, Díaz, 2022). This approach, combined with molecular methods for identifying parental forms and analyzing hybrid progeny, has made it possible to obtain a large number of high-yielding varieties and hybrids of crops, including maize hybrids with a high content of provitamin A (Pixley et al., 2013; Muthusamy et al., 2014; Liu et al., 2015; Menkir et al., 2017; Prasanna et al., 2020).

In this regard, of interest is the molecular screening of collections of various crops, which makes it possible to determine allelic variants of genes, new alleles, and the linkage of alleles with morphophysiological characteristics (Langridge, Fleury, 2011; Pasala, Pandey, 2020). From a scientific point of view, the totality of the results obtained contributes to a more accurate understanding of the function of specific genes. At the same time, screening of collections is an important stage of breeding, as it allows to assess the representation in the breeding material of a specific allelic variant that determines the desired economically important trait, as well as to identify donors of this trait for introduction into the breeding process (Langridge, Fleury, 2011).

In this work, accessions of 165 maize inbred lines of domestic breeding were characterized by the 5ʹ-UTR allelic variant of the LCYE gene. The activity of lycopene-ɛ-cyclase is considered to be inversely related to the biogenesis of β-carotene and the corresponding β,β-xanthophylls, which, in turn, determines the color of the grain (pale yellow and orange indicate a shift towards β,ε- and β,β-branches (see Fig. 1), respectively) (Harjes et al., 2008; Babu et al., 2013; Zunjare et al., 2018). A decrease in LCYE gene activity and, as a result, significant changes in the ratio of β,ε- and β,β-carotenoids are closely associated with mutations in the 5ʹ-UTR region of the gene, namely, with insertions of transposable elements near the translation initiation point (alleles A1 and A4) (Harjes et al., 2008). At the same time, the highest efficiency of provitamin A accumulation in grain is linked to the A4 allele (Babu et al., 2013; Zunjare et al., 2018). Given this, molecular markers have been developed to identify various allelic variants of the 5ʹ-UTR sequence of the LCYE gene (Harjes et al., 2008; Babu et al., 2013). Screening of Z. mays collections using these markers made it possible to identify donors of the A4 allele and introduce them into breeding programs to obtain maize lines and hybrids with a high content of provitamin A (Harjes et al., 2008; Babu et al., 2013).

Our analysis of 165 inbred lines did not reveal accessions carrying the A4 allele linked to the enhanced accumulation of provitamin A. This indicates that for biofortification for an increased content of provitamin A, sources other than the lines of this collection should be involved. However, in addition to the A2 allele, screening detected two other variants of the 5ʹ-UTR region, the A5 allele (Arkhestova et al., 2023), as well as the previously undescribed A6 allele (see Fig. 3).

Next, we tested the possibility of a relationship between the 5ʹ-UTR LCYE allele variant (A2, A5, or A6) and the level of LCYE expression. Photosynthetic tissues of seedlings were used for the analysis because data on the correlation of LCYE alleles with the content of carotenoids in corn are limited mainly to grain, and also due to the predominant cultivation of corn for silage in Russia, since the presence of such a correlation can serve as the basis for identifying donors of the trait of increased biosynthesis provitamin A in maize photosynthetic tissue (silage). In the case of a clear association of any allele with the level of LCYE gene expression, donors of this allele could be used in the breeding of silage corn with a high content of provitamin A.

qRT-PCR was performed on 14 accessions out of 165 lines studied in this work. Among these 14 lines, all three alleles of the 5ʹ-UTR LCYE (A2, A5, and A6) were present. Since the result showed no association of the detected 5ʹ-UTR LCYE allelic variants with the level of LCYE expression in the leaf (see Fig. 4), it can be assumed that there is no such association for the rest of the analyzed collection. The absence of the desired dependence can partly be explained by the fact that the ratio of the amount of synthesized β,ε- and β,β-carotenoids depends on the level of expression of not only LCYE, but also the gene of lycopene-β-cyclase LCYB (Bai et al., 2009) or other carotenogenesis genes, for example, phytoene synthase genes (PSY) (Orlovskaya et al., 2016).

qRT-PCR data showed a clear division of accessions into two groups – with high and low expression of LCYE. Considering the known antioxidant role of xanthophylls in plant photoprotection (Jahns, Holzwarth, 2012), it can be assumed that in 10 lines with low LCYE expression (see Fig. 4), such protection is carried out mainly by carotenoids of the main xanthophyll cycle (β,β-branch). At the same time, in the remaining 4 lines with high LCYE expression, presumably synthesizing significant amounts of β,ε-carotenoids, photoprotection can actively involve an additional lutein-5,6-epoxide cycle (β,ε-branch).

It is also possible that lines with low LCYE expression (see Fig. 4) and a presumed shift of the carotenoid biosynthetic pathway towards the β,β-branch synthesize relatively more phytohormones (strigolactones and abscisic acid) produced by the action of carotenoid-cleaving dioxygenases (Dhar et al., 2020). Thus, ABA is formed by 9-cis-epoxycarotenoid dioxygenases NCED from 9-cis-violaxanthin and 9-cisneoxanthine (violaxanthin derivatives), while strigolactones are synthesized by cleavage of β-carotene by CCD dioxygenases (Nambara, Marion-Poll, 2005; Cutler et al., 2010; Dhar et al., 2020). ABA plays a crucial role in the adaptability of plants, including Z. mays, to various environmental conditions, mediating growth, development, stress response, and nutrient distribution (Huang et al., 2017; Yue et al., 2021). Strigolactones are actively involved in the stress response of plants (López-Ráez et al., 2010). In view of the above, the expected increased synthesis of apocarotenoids may indicate greater adaptability of maize lines with low LCYE expression in the vegetative tissue.

## Conclusion

In this work, we analyzed variants of the 5′-UTR allele of the lycopene-ɛ-cyclase LCYE gene in the genome of 165 inbred maize lines of Russian selection. As a result, three groups of accessions carrying the A2 (64 lines), A5 (31), or A6 (70) alleles were identified. The shortest of them, the A5 allele, differed by one and two deletions from A6 and A2, respectively. To assess the possible dependence of the LCYE mRNA level in leaves on the 5′-UTR allelic variant, gene expression was determined in 14 lines differing in allelic variants. Based on the data obtained, it can be argued that the desired associations are absent. We assume that maize lines with low expression of the LCYE gene can serve as a source of traits of increased plant stress resistance and enhanced synthesis of provitamin A in photosynthetic tissue. In this case, the marker will not be the 5′-UTR LCYE allelic variant, but the level of expression of the LCYE gene. Confirmation of this possibility will require further studies on a larger number of accessions.

## Conflict of interest

The authors declare no conflict of interest.
